# Prevalence and Sociodemographic Correlates of Behavioral Addictions Among Medical Students: A Cross-Sectional Study

**DOI:** 10.1192/j.eurpsy.2026.10874

**Published:** 2026-07-31

**Authors:** S. Jaswal, S. Malhotra

**Affiliations:** psychiatry, indira gandhi medical college, shimla, India

## Abstract

**Introduction:**

Behavioral addictions are emerging mental health concerns, especially among young adults, including medical students. In ICD-11, behavioral addictions are recognized under the broader category of “Disorders due to addictive behaviors”, which marks a significant evolution from ICD-10. These addictions often coexist with elevated stress levels. Among medical students, who frequently face high academic pressure, sleep deprivation, and social isolation, the risk of developing such maladaptive coping mechanisms is particularly pronounced. As such, understanding the prevalence, risk factors, and clinical impact of behavioral addictions in this vulnerable group is crucial.

**Objectives:**

To estimate the prevalence and severity of behavioral addictions (internet addiction, internet gaming disorder, problematic pornography use, and gambling) among undergraduate medical students and examine their association with stress and sociodemographic factors.

**Methods:**

A cross-sectional study was conducted among undergraduate MBBS students. An online questionnaire was circulated amongst the students which included a sociodemographic profile performa along with Young’s Internet Addiction Test (IAT), Internet Gaming Disorder Scale-Short Form (IGDS9-SF), Problematic Pornography Consumption Scale (PPCS), Problem Gambling Severity Index (PGSI), and Perceived Stress Scale (PSS).Data was collected and descriptive statistics and Chi-square tests were applied for analysis using Epi Info 7.2.6.

**Results:**

A total of 480 students were approached for the study out of which 260 students completed the questionnaires. The study sample included 48.5% male and 51.5 % females. Maximum respondents belonged to the age range of 21-25 years Substance use was present in 19.6%. Internet Gaming Addiction was present in 50.4% of students, significantly higher in males (61.9%) compared to females (39.6%) (p < 0.001). Problematic Pornography Use was found in 5.0% of students, significantly higher among males (9.5%) than females (0.7%) (p = 0.001). Problem Gambling prevalence was 27.3%, significantly associated with male gender and age group (p < 0.001). Internet Addiction was observed in 51.9%. Internet Gaming Disorder (IGD) was diagnosed in 2.7% of students. Moderate and high stress were reported in 73.5% and 18.1%, respectively. However, no significant correlation was found between stress levels and specific behavioral addictions.

**Conclusions:**

Behavioral addictions, especially internet gaming and gambling, are highly prevalent among medical students, with male gender showing greater vulnerability. Although stress was common, its direct association with specific addictions was not statistically significant. These findings highlight the need for awareness programs and preventive interventions targeting behavioral addictions in medical student populations.

**Disclosure of Interest:**

None Declared

